# Dynamics of Antimicrobial Resistance Carriage in Koalas (*Phascolarctos Cinereus*) and Pteropid Bats (*Pteropus Poliocephalus*) Before, During and After Wildfires

**DOI:** 10.1007/s00248-024-02351-w

**Published:** 2024-02-09

**Authors:** Fiona K. McDougall, Natasha Speight, Oliver Funnell, Wayne S. J. Boardman, Michelle L. Power

**Affiliations:** 1 School of Natural Sciences, Faculty of Science and Engineering, Macquarie University, Sydney, NSW 2109 Australia; 2 School of Animal and Veterinary Sciences, Faculty of Sciences, Engineering and Technology, University of Adelaide, Roseworthy, SA 5371 Australia; 3 Zoos South Australia, Frome Rd, Adelaide, SA 5001 Australia

**Keywords:** Class 1 Integron, *bla*TEM, Bushfires, Conservation, Flying fox, Fruit bat, One health

## Abstract

In the 2019–2020 summer, wildfires decimated the Australian bush environment and impacted wildlife species, including koalas (*Phascolarctos cinereus*) and grey headed flying fox pups (Pteropid bats, *Pteropus poliocephalus*). Consequently, hundreds of koalas and thousands of bat pups entered wildlife hospitals with fire-related injuries/illness, where some individuals received antimicrobial therapy. This study investigated the dynamics of antimicrobial resistance (AMR) in pre-fire, fire-affected and post-fire koalas and Pteropid bat pups. PCR and DNA sequencing were used to screen DNA samples extracted from faeces (koalas and bats) and cloacal swabs (koalas) for class 1 integrons, a genetic determinant of AMR, and to identify integron-associated antibiotic resistance genes. Class 1 integrons were detected in 25.5% of koalas (68 of 267) and 59.4% of bats (92 of 155). Integrons contained genes conferring resistance to aminoglycosides, trimethoprim and beta-lactams. Samples were also screened for *bla*TEM (beta-lactam) resistance genes, which were detected in 2.6% of koalas (7 of 267) and 25.2% of bats (39 of 155). Integron occurrence was significantly higher in fire-affected koalas in-care compared to wild pre-fire koalas (*P* < 0.0001). Integron and *bla*TEM occurrence were not significantly different in fire-affected bats compared to pre-fire bats (*P* > 0.05), however, their occurrence was significantly higher in fire-affected bats in-care compared to wild fire-affected bats (*P* < 0.0001 and *P* = 0.0488 respectively). The observed shifts of AMR dynamics in wildfire-impacted species flags the need for judicious antibiotic use when treating fire-affected wildlife to minimise unwanted selective pressure and negative treatment outcomes associated with carriage of resistance genes and antibiotic resistant bacteria.

**Supplementary Information:**

The online version contains supplementary material available at 10.1007/s00248-024-02351-w.

## Introduction

Antimicrobial resistance (AMR) is now widespread in diverse wildlife species [1, 2]. The past decade has seen a rapid increase in reports of antimicrobial resistant bacteria in wildlife [3], including in Australian wild mammals and birds [4–10]. The carriage of antimicrobial resistant bacteria by wildlife poses a risk to their health when an individual requires antibiotic treatment, potentially leading to negative treatment outcomes associated with antibiotic resistant bacterial pathogens [11–13]. For some wildlife species, the carriage of AMR is higher is animals which have entered veterinary hospitals and rehabilitation environments compared to their free-living counterparts [8, 14, 15]. A higher prevalence of AMR in wildlife in captive settings (both wild animals in-care and wildlife in zoological collections) has been associated with multiple factors including, the administration of antibiotics, increased exposure to antimicrobial resistant bacteria and increased transmission of resistant bacteria between co-housed individuals in-care [8, 15–17].

The rise of AMR can, in part, be attributed to the rapid spread of mobile genetic elements (MGE), such as plasmids and transposons, which carry diverse antimicrobial resistance genes (ARG) [18]. MGE facilitate the horizontal transfer of ARG and associated genetic mechanisms between bacteria of different strains and species [18]. The class 1 integron is a genetic mechanism which has played a significant role in the dissemination of AMR [19], particularly in Gram-negative bacterial pathogens [20]. The class 1 integron has key components that enable it to capture, integrate and express genes that confer resistance to a diverse range of antibiotics [21]. Class 1 integrons are comprised of an integrase gene (*intl1*), a promoter (*Pc*), a variable number of gene cassettes encoding ARGs, which form a gene cassette array, and typically a 3’-conserved segment (*qacEdelta*) [21]. Alternatively, class 1 integrons may have an IS*26* transposase in place of the typical *qacEdelta* [22].

The class 1 integron has been proposed as an indicator of anthropogenic DNA pollution in the environment and subsequently, in wildlife [23]. Wildlife in habitats located within close proximity to humans and/or domestic animals typically have a higher prevalence of class 1 integrons compared to wildlife living in remote natural environments [24, 25]. In Australian wildlife, the class 1 integron has been detected in diverse species, including the grey-headed flying fox (*Pteropus poliocephalus*) [14], the little penguin (*Eudyptula minor*) [26], the brush-tailed rock-wallaby (*Petrogale penicillate*) [25], the Australian sea lion (*Neophoca cinerea*) and the Australian fur seal (*Arctocephalus pusillus doriferus*) [4], indicating that antibiotic resistant bacteria are well integrated in wildlife gut microbiomes.

Beta-lactam antibiotics, including penicillin, amoxicillin and cephalosporins, are the most abundant antibiotic class administered to humans [27], domestic animals [28], and also commonly used in wildlife medicine [29]. Resistance to beta-lactam antibiotics is predominantly associated with the production of beta-lactamases, of which, *bla*TEM is one of the most common found in Gram-negative bacteria [30]. Along with class 1 integrons, the *bla*TEM gene can be used as an indicator of anthropogenic AMR in the environment [31]. The *bla*TEM gene has been detected in Australian wildlife species, including the grey-headed flying fox (*P. poliocephalus*) [6, 7] and Australian silver gulls (*Chroicocephalus novaehollandiae*) [5].

In the summer of 2019–2020, catastrophic wildfires extended across south-eastern Australia, resulting in the destruction of almost 12.6 million hectares [32, 33] and the loss of hundreds of millions of native animals [33, 34]. In addition to the millions of native animals directly lost or impacted by the wildfires (primary fire-affected wildlife), millions of animals were also displaced, faced habitat loss, food and water shortages, or acquired other injuries (secondary fire-affected wildlife) [34]. Native wildlife species impacted by the 2019–2020 fires included mammals (inclusive of marsupials and monotremes), reptiles, frogs and birds [34]. The 2019–2020 wildfires in Australia are part of a wider global fire threat for biodiversity and wildlife arising from climate change [35, 36].

The koala (*Phascolarctos cinereus*), an iconic Australian arboreal marsupial, was one of the most heavily impacted species in the 2019–2020 wildfires [34, 37–40]. In South Australia, approximately 280,000 hectares were burnt and an estimated 40,000 to 50,000 koalas died [41], with the vast majority of losses occurring on Kangaroo Island, South Australia [34]. Although koalas in South Australia are not listed as a threatened species, unlike the endangered eastern populations [42], it is estimated that up to 85% of the Kangaroo Island koala population was lost in the 2019–2020 wildfires [34]. Several hundred primary and secondary fire-affected koalas which survived the fires, were taken into care on Kangaroo Island and on the South Australian mainland, and treated for burns, injuries, disease, starvation and/or dehydration [34, 37].

The grey headed flying fox (*P. poliocephalus*) is an endemic Pteropid bat species found across eastern Australia [43]. They roost in trees located in both urban and forested areas, and typically form colonies containing up to 50,000 individuals [43]. Grey headed flying foxes (hereon after bat) are highly mobile, flying long distances to forage each night (> 50 km) and travelling hundreds of kilometres between colonies [44]. Each year, bat pups are born between October and December, and spend the first month of life carried by their mothers [45]. After this time, pups remain in the bat roost at night, while their mothers fly out to forage, until they are old enough to forage for themselves (3–4 months) [45, 46]. Grey headed flying foxes are listed as vulnerable to extinction on the IUCN Red List [47], and prior to the 2019–2020 wildfires they were already being negatively impacted by habitat loss, drought, extreme heat events and disease [43]. During the 2019–2020 wildfires, an unknown number of bats were killed or directly impacted by wildfires (i.e. primary fire-affected bats) [34, 48]. A large number, estimated to be > 70,000 bats, were lost due to fire-related impacts including pup abandonment [49], habitat loss and food shortages [34], termed secondary fire-affected bats. During the 2019–2020 wildfires, over 2,500 bat pups and adults were rescued and taken into care until they were deemed suitable for release back into wild colonies [49].

This study aimed to investigate the impact of fire on the dynamics of AMR determinants in fire-affected wildlife. The occurrence and diversity of class 1 integrons and *bla*TEM genes in DNA samples from pre-fire, fire-affected and post-fire koalas (faecal and cloacal samples) and bat pups (faecal samples) were assessed. Additionally, the study aimed to evaluate the association of class 1 integron and *bla*TEM carriage with fire-season status, entry into care and antibiotic administration.

## Methods

### Koala Faecal Sampling and DNA Extraction

Faecal samples (*n* = 190) were opportunistically collected from wild-caught koalas at three sites (Cleland Conservation Park, Morialta Conservation Park and Belair National Park) located within the Mount Lofty Ranges, South Australia, and provided for this study (Supplementary Data 1). These three sites (Cleland Conservation Park, Morialta Conservation Park and Belair National Park) are unfenced wild koala habitat located 10–12 km from Adelaide city centre. Koalas in Cleland and Morialta Conservation Parks were sampled once (April 2018) and koalas in the Belair National Park were sampled twice (April 2018 and October 2022) (Supplementary Data 1). Relative to the 2019–2020 wildfires, the 190 samples were divided into pre-fire samples (*n* = 91, collected April 2018) and post-fire samples (*n* = 99, collected October 2022) (Supplementary Data 1). Faecal samples were obtained by either rectal swab (*n* = 113) (COPAN, Brescia, Italy) or from faecal pellets (*n* = 77) (Supplementary Data 1). Samples were stored at 4˚C until transferred to the laboratory, then frozen at -30˚C until processing. DNA was extracted from faecal samples using the ISOLATE II Fecal DNA Kit (Bioline, London, UK). Metadata was provided for all individual koalas (Supplementary Data 1).

### Female Koala Cloacal Swab DNA Samples

A subset of samples were provided as DNA extractions that were derived from female koala cloacal swab samples (*n* = 81) collected from koalas on Kangaroo Island, between 2014 and 2017 (represented pre-fire samples) and during the 2019–2020 wildfires (represented fire-affected samples) (Supplementary Data 1). The pre-fire DNA samples (*n* = 49) were from wild female koalas caught as part of a koala sterilisation program run by the South Australian Department for Environment and Water (DEW) between 2014 and 2017 [50] (Supplementary Data 1). The fire-affected samples (*n* = 32) were obtained from previously wild female koalas that entered into veterinary care during the 2019–2020 wildfires [37] (Supplementary Data 1). All samples were stored at -80˚C or -30˚C. Metadata was provided for all individual koalas (Supplementary Data 1).

### Grey Headed Flying Fox Faecal Sampling and DNA Extraction

Bat faecal samples (*n* = 155) were collected from pups at three locations in New South Wales (Sydney, Shoalhaven and Bega) and one location in South Australia (Adelaide) (Supplementary Data 1). Bat pups in Shoalhaven and Bega were sampled once (during one birthing season), and pups in Adelaide and Sydney were sampled twice (during two different birthing seasons) (Supplementary Data 1). Relative to the 2019–2020 wildfires, the 155 samples were classified as pre-fire samples (*n* = 77, collected October 2018 to April 2019), fire-affected samples (*n* = 48, collected November 2019 to February 2020), and post-fire samples (*n* = 30, collected January 2021) (Supplementary Data 1). Most of the sampled bat pups were in-care (118 of 155) and the remaining were wild (37 of 155) (Supplementary Data 1). The FecalSwab™ system (COPAN, Brescia, Italy) was used to collect all faecal samples, which included faecal deposits from 116 live bat pups and faecal material from the intestines of 39 deceased pups that had been stored at -20˚C until necropsy. Metadata is provided for all individual bat pups (Supplementary Data 1). FaecalSwab samples were frozen at -30˚C until processing. DNA was extracted from FecalSwab media using the ISOLATE II Fecal DNA Kit (Bioline, London, UK) and stored at -30˚C.

### 16 S rRNA PCR Screening of DNA Samples

All DNA samples (*n* = 426) underwent a 16 S rRNA PCR to confirm DNA competency prior to inclusion in screening for integrons and *bla*TEM genes. The 16 S rRNA PCR was performed using the universal eubacterial primers f27 and r1492 [51] as previously described [52]. Only samples which amplified in the 16 S rRNA PCR, and deemed PCR-competent, were utilised in this study (Supplementary Data 1).

### PCR Screening for Class 1 Integrons

All faecal and cloacal swab DNA samples deemed PCR-competent were then screened for the presence of class 1 integrons. Primers HS463a and HS464 [53] were used to target the class 1 integron integrase gene (*intI1*) using using GoTaq® Colourless Master Mix (Promega, Madison, WI, USA) and PCR conditions as previously described [14]. All PCRs included an *intI1* positive control sample (*E. coli* strain KC2) [19], and a negative control (PCR-grade H_2_O), and were visualised using gel electrophoresis.

### PCR Screening for Gene Cassette Arrays

Samples identified as *intI1* positive were then screened using two further PCRs to detect gene cassette arrays and identify ARGs. Two primers sets were used to target gene cassette arrays with either the conserved 3’ terminus (primers HS458 and HS459) [54] or the alternate IS*26* transposase 3’ terminus (primers HS458 and JL-D2) [22]. Both the HS458/HS459 and HS458/JL-D2 PCRs used GoTaq® Colourless Master Mix (Promega, Madison, WI, USA) and the same PCR conditions, as previously described for HS458/HS459 [14]. All PCRs included a positive control sample; *E. coli* strain KC2 as the HS458/459 positive control [19] or *E. coli* strain FF993W as the HS458/JL-D2 positive control [6], and a negative control (PCR-grade H_2_O), and PCR products were visualised using gel electrophoresis.

Where a single amplicon was present, PCR products were purified using the MinElute PCR Purification Kit (Qiagen, Hilden, Germany). Purified PCR products underwent DNA sequencing at The Ramaciotti Centre for Genomics (University of New South Wales, Sydney, Australia) using Big Dye Terminator chemistry version 3.1 and ABI 3730/3730 × 1 Capillary Sequencers (Applied Biosystems, Foster City, CA, USA). DNA sequences were analysed using Geneious Prime software (versions R11 to 2023.1.2; Biomatters Limited, Auckland, New Zealand) and gene cassette arrays were identified using BLASTN searches (https://blast.ncbi.nlm.nih.gov/Blast.cgi) and ARG variants confirmed using ResFinder 4.1 (available at http://www.genomicepidemiology.org/services/) [55]. Where multiple PCR amplicons were present, bright gel bands were excised and purified using the QIAquick Gel Extraction Kit (Qiagen, Hilden, Germany) and then sequenced, analysed and ARGs identified as described above for single amplicon HS458/HS459 PCR products. Where gene cassette arrays were not observed in *IntI1* positive samples, HS463a/HS464 PCR products were purified, sequenced, and analysed as described above for HS458/HS459 PCR products. DNA sequences were confirmed as *intI1* genes using BLASTN searches (https://blast.ncbi.nlm.nih.gov/Blast.cgi).

### **PCR Screening for*bla*TEM Genes**

All faecal and cloacal swab DNA samples deemed to be PCR-competent were also screened for the presence of *bla*TEM genes using the primers TEM-C 5’TCGGGGAAATGTGCGCG3’ and TEM-D 5’TGCTTAATCAGTGAGGCACC3’ [56].

The *bla*TEM PCRs used TopTaq DNA Polymerase (Qiagen, Hilden, Germany) and cycling conditions of 94 °C 3 min; 35 cycles of 94 °C 30 s, 50 °C 20 s, 72 °C 2 min; 72 °C 5 min. All PCRs included a *bla*TEM-1B positive control sample (*E. coli* strain FF993W) [6] and a negative control (PCR-grade H_2_O), and were visualised using gel electrophoresis. The *bla*TEM PCR products were purified, sequenced, analysed and *bla*TEM genes confirmed as described above for *intI1* PCR products. To identify *bla*TEM gene variants, representative sequences were uploaded to ResFinder 4.1 (available at http://www.genomicepidemiology.org/services/ and accessed in May 2023) [55].

### **Class 1 Integron and*bla*TEM Sequence Annotation and Accessions**

Representative sequences for *intI1*, gene cassette arrays and *bla*TEM genes were manually annotated in Geneious Prime software (version 2023.1.2; Biomatters Limited, Auckland, New Zealand) using reference sequences identified in BLASTN searches (https://blast.ncbi.nlm.nih.gov/Blast.cgi) and uploaded to GenBank under accession numbers OR095837 - OR095843 and OR095845 - OR095856 (Supplementary Data 1).

### Statistical Analysis

Fisher’s exact test (two-tailed) was used to evaluate any statistical difference in the occurrence of *intI1* and *bla*TEM genes in paired samplings collected from the same locations at different time points (i.e., pre-fire/fire-affected/post-fire), from different environments (i.e., wild and in-care), and with or without antibiotic administration. Calculations were performed using GraphPad (available at https://www.graphpad.com), with *P* < 0.05 indicating statistical significance.

## Results

### Detection of Class 1 Integrons in Koalas and Bats

The *intI1* gene, signalling the presence of the class 1 integron, was detected in 25.5% of all PCR-competent koala DNA samples (68 of 267), and ranged from 0.0 to 46.9% across the six sampling events (Table 1). In Mount Lofty Ranges koalas, integron frequency ranged from 6.9 to 43.6% and from 0.0 to 46.9% in Kangaroo Island koalas (Table 1). In bat pups, the *intI1* gene was detected in 59.4% of all PCR-competent DNA samples (92 of 155) and ranged from 23.3 to 100% across the six sampling events (Table 1).

A total of 46 class 1 integrons containing ARGs were identified in koalas and bats, which were differentiated into integrons with the conserved 3’ terminus (*qacEdelta*; *n* = 27) and those with the alternate IS*26* transposase 3’ terminus (IS*26; n* = 19) (Table 1). Ten distinct types of class 1 integrons carrying ARGs were identified in both wildlife hosts, with four types detected in koala DNA samples and eight types in bat DNA samples (Table 1). The number of gene cassettes in the class 1 integrons from koalas and bats ranged from one to four (Table 1).

Twelve different ARGs and two hypothetical proteins (*ORFD* and *ORFX*) were identified (Table 1). The majority of integron ARGs conferred resistance to either aminoglycosides (*aadA/aadB*, *n* = 6), trimethoprim (*dfrA*, *n* = 4), beta-lactams (*OXA-2*, *n* = 1) or quaternary ammonium compounds (*qacL*, *n* = 1) (Table 1). In koalas, *dfrA5/qacEdelta* was the most frequent class 1 integron identified (7 of 10) and *dfrA17/aadA5/IS26* was the most frequent class 1 integron in bat pups (18 of 36) (Table 1). Four bat pup samples carried two different class 1 integrons containing ARGs, *aadA2/qacEdelta* plus *dfrA17/aadA5/*IS26 (*n* = 3) and *aadA2/qacEdelta* plus *dfrA5/qacEdelta* (*n* = 1) (Supplementary Data 1).

For one integron (*aadB/ORFX/OXA-2----aadA1/qacEdelta)* the full-length sequence was not obtained due to its long length (~ 3000 bp), with both partial sequences, i.e. the forward sequence (1,164 bp, *aadB/ORFX/OXA-2*) and the reverse sequence (1,115 bp, *aadA1/qacEdelta*), returning a 100% match to a class 1 integron (*intI1/aadB/ORFX/OXA-2/aadA1/qacEdelta* ) from a *Corynebacterium amycolatum* isolate (GenBank accession AJ871915) (Supplementary Data 1). A BLASTN search of the single *ORFX* gene resulted in only one match (100% identity), also from the *C. amycolatum* isolate (GenBank accession AJ871915).

There were 14 samples that were *intI1* positive but no ARGs were present in the cassette array. A further 26 samples that were confirmed as *intI1* positive by DNA sequencing, either failed to amplify in both the HS458/459 and HS458/JL-D2 PCRs (*n* = 16), or sequencing identified non-specific PCR products (i.e., non-class 1 integrons) (*n* = 10). Multiple bands were observed in the HS458/459 and/or HS458/JL-D2 PCRs for 78 *intI1* positive samples, in which, cassette array sequencing was not feasible (Supplementary Data 1).

The closest GenBank matches for representative *intI1* and class 1 integron sequences from koala and bat DNA samples showed between 99.4% and 100% identity to GenBank accessions (Supplementary Data 1).

### **Detection of*bla*TEM Genes in Koala and Bat DNA Samples**

The *bla*TEM gene was detected in 2.6% of all PCR-competent koala DNA samples (7 of 267), with all detections occurring in 2018 at two Mount Lofty Ranges locations (Cleland and Morialta) (Table 1). Of the two koala sampling events where *bla*TEM was detected, the occurrence ranged from 10.3 to 13.0%, and two *bla*TEM gene variants were identified, namely *bla*TEM-116 and *bla*TEM-1 C (Table 1).

In bat pups, the *bla*TEM gene was detected in 25.2% of all PCR-competent DNA samples (39 of 155), with detections occurring at all sampled locations, except Bega (Table 1). Of the five bat sampling events where *bla*TEM was detected, the occurrence ranged from 6.7 to 47.4% (Table 1). Three *bla*TEM gene variants were identified, with *bla*TEM-1B being the most frequent (21 of 39) (Table 1). The identity of the *bla*TEM gene variant could not be determined in 12 samples due to sequence data having multiple peaks (indicative of multiple variants) or insufficient sequence length (Table 1).

The closest GenBank matches for representative *bla*TEM gene sequences from koala and bat pup DNA samples showed between 99.9% and 100% identity to GenBank accessions (Supplementary Data 1).

**Table 1:** Class 1 integrons and *bla*TEM genes detected in koala faecal and cloacal swab DNA and in grey headed flying fox (Pteropid bat) pup faecal DNA from samples collected before, during and after the 2019–2020 wildfires. Koalas were located in the Mount Lofty Ranges (MLR), South Australia (SA) and on Kangaroo Island, South Australia (SA). Grey headed flying fox pups were from multiple locations in New South Wales (NSW) and South Australia (SA).

Sampling location	Sampling event	Fire season status	In-care/Wild/ Wild-caught	% intI1 positive	Cassette arrays containing ARGs	No. identified	% blaTEM positive	blaTEM variant	No. identified
**Koala faecal DNA samples**									
Cleland, MLR, SA	9–11 Apr 2018	Pre-fire	Wild-caught	43.6 (17/39)	*dfrA5/qacEdelta*	7	10.3 (4/39)	*bla*TEM-116	4
Morialta, MLR, SA	13–14 Apr 2018	Pre-fire	Wild-caught	8.7 (2/23)	Not identified	*–*	13.0 (3/23)	*bla*TEM-1 C	3
Belair, MLR, SA	12 Apr 2018	Pre-fire	Wild-caught	6.9 (2/29)	Not identified	*–*	0.0 (0/29)	*–*	*–*
Belair, MLR, SA	Oct 2022	Post-fire	Wild-caught	32.7 (32/98)	Not identified	*–*	0.0 (0/98)	*–*	*–*
Koala cloacal swab DNA samples									
Kangaroo Island, SA	2014–2017	Pre-fire	Wild-caught	0.0 (0/46)	*–*	*–*	0.0 (0/46)	*–*	*–*
Kangaroo Island, SA	Jan - Mar 2020	Fire-affected	In-care	46.9 (15/32)	*aadA2*/*qacEdelta*	1	0.0 (0/32)	*–*	*–*
					*dfrA14*/IS*26*	1			
					*dfrA33-qacEdelta*	1			
All koala DNA samples				25.5(68/267)		10	2.6 (7/267)		7
**Grey headed flying fox (Pteropid bat) pup faecal DNA samples**									
Shoalhaven, NSW	Oct 2018 - Jan 2019	Pre-fire	In-care	100 (6/6)	Not identified	-	33.3 (2/6)	*bla*TEM-1B	2
Adelaide, SA	Nov 2018 - Jan 2019	Pre-fire	In-care	84.6 (44/52)	*aadA2/qacEdelta*	5	46.2 (24/52)	*bla*TEM-1B	6
					*aadA9/qacEdelta*	1		*bla*TEM-33	4
					*dfrA5/qacEdelta*	1		*bla*TEM-116	2
					*dfrA17/aadA5*/IS*26*	18		*bla*TEM-ND	12
Adelaide, SA	Jan 2021	Post-fire	In-care	23.3 (7/30)	*dfrA5/qacEdelta*	1	6.7 (2/30)	*bla*TEM-1B	2
Sydney, NSW	Jan 2019 - Apr 2019	Pre-fire	In-care	78.9 (15/19)	*aadA2/qacEdelta*	2	47.4 (9/19)	*bla*TEM-1B	9
					*qacL/qacEdelta*	1			
Sydney, NSW	Jan 2020 - Feb 2020	Fire-affected	In-care	100 (11/11)	*aadA1/qacEdelta*	2	18.2 (2/11)	*bla*TEM-1B	2
					*aadA2/qacEdelta*	2			
					*aadA4/ORFD/qacEdelta*	1			
					*aadB/ORFX/OXA-2---* *---aadA1/qacEdelta*	1			
					*dfrA5/qacEdelta*	1			
Bega, NSW	Nov 2019 - Feb 2020	Fire-affected	Wild	24.3 (9/37)	Not identified	*–*	0.0 (0/37)	*–*	*–*
All grey headed flying fox (Pteropid bat) DNA samples				59.4 (92/155		36	25.2 (39/155)		39

### **Distribution of Class 1 Integrons and*bla*TEM Genes Across Koala and Bats**

**Fig. 1:**
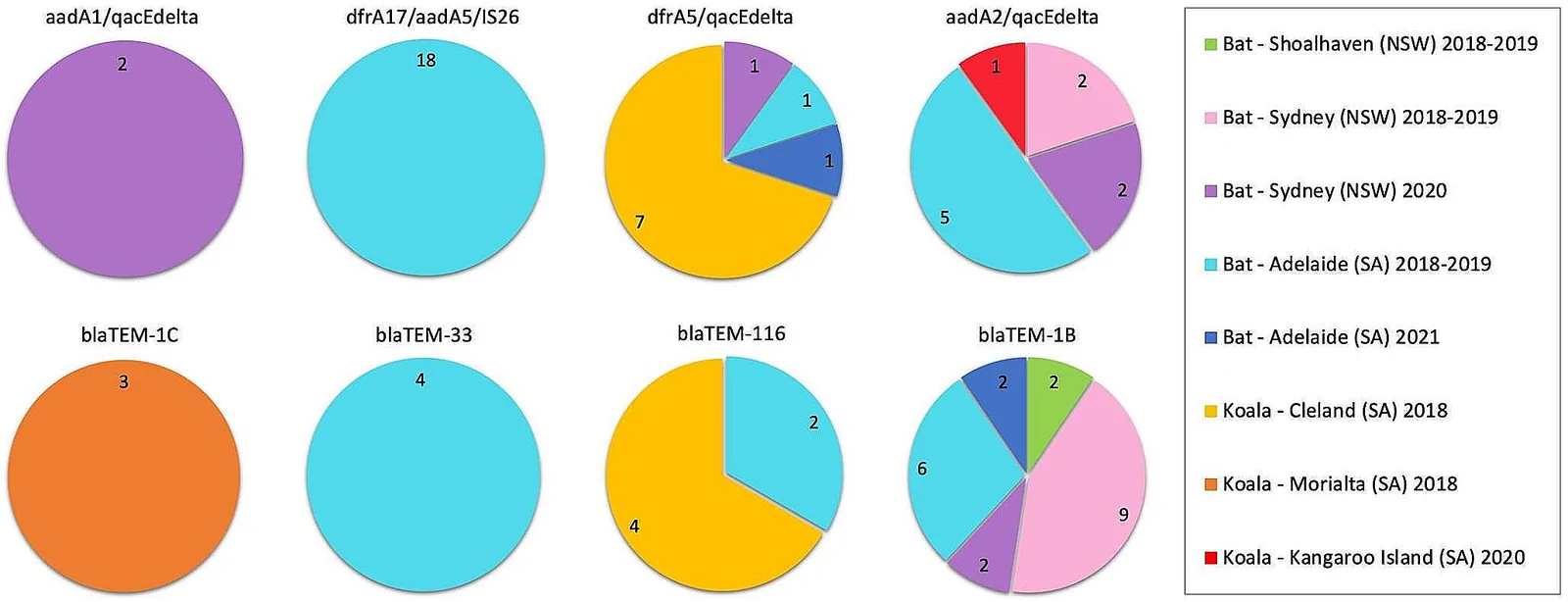
The distribution of four class 1 integron gene cassette arrays and four *bla*TEM genes that were detected in two or more samples from koalas and/or Pteropid bat pups. NSW, New South Wales. SA, South Australia

Across all koala and bat samples, 10 distinct class 1 integrons and four distinct *bla*TEM gene variants were found, of which, eight (four class 1 integrons and all four *bla*TEM gene variants) were detected in two or more samples, and the remaining six class 1 integrons were detected in only one sample (Table 1; Fig. 1). Of the eight integrons and *bla*TEM genes detected more than once, four were detected in samples from multiple sampling events (three in both koalas and bats, and one in bats only), and the remaining four were exclusively found in samples from one sampling event (one in koalas and three in bats) (Fig. 1).

The *bla*TEM-1B gene was most widely distributed and detected in samples from five of six bat sampling events (Fig. 1). The integron arrays *aadA2/qacEdelta* and *dfrA5/qacEdelta* were the next most frequent and detected in samples from four sampling events (Fig. 1). The *dfrA17/aadA5/IS26* integron was detected in 18 samples, however, distribution was restricted to bats from one sampling event (Adelaide 2018–2019) Fig. 1).

### **Geographical Clustering of Class 1 Integrons and*bla*TEM Genes in Mount Lofty Ranges Koalas**

**Fig. 2:**
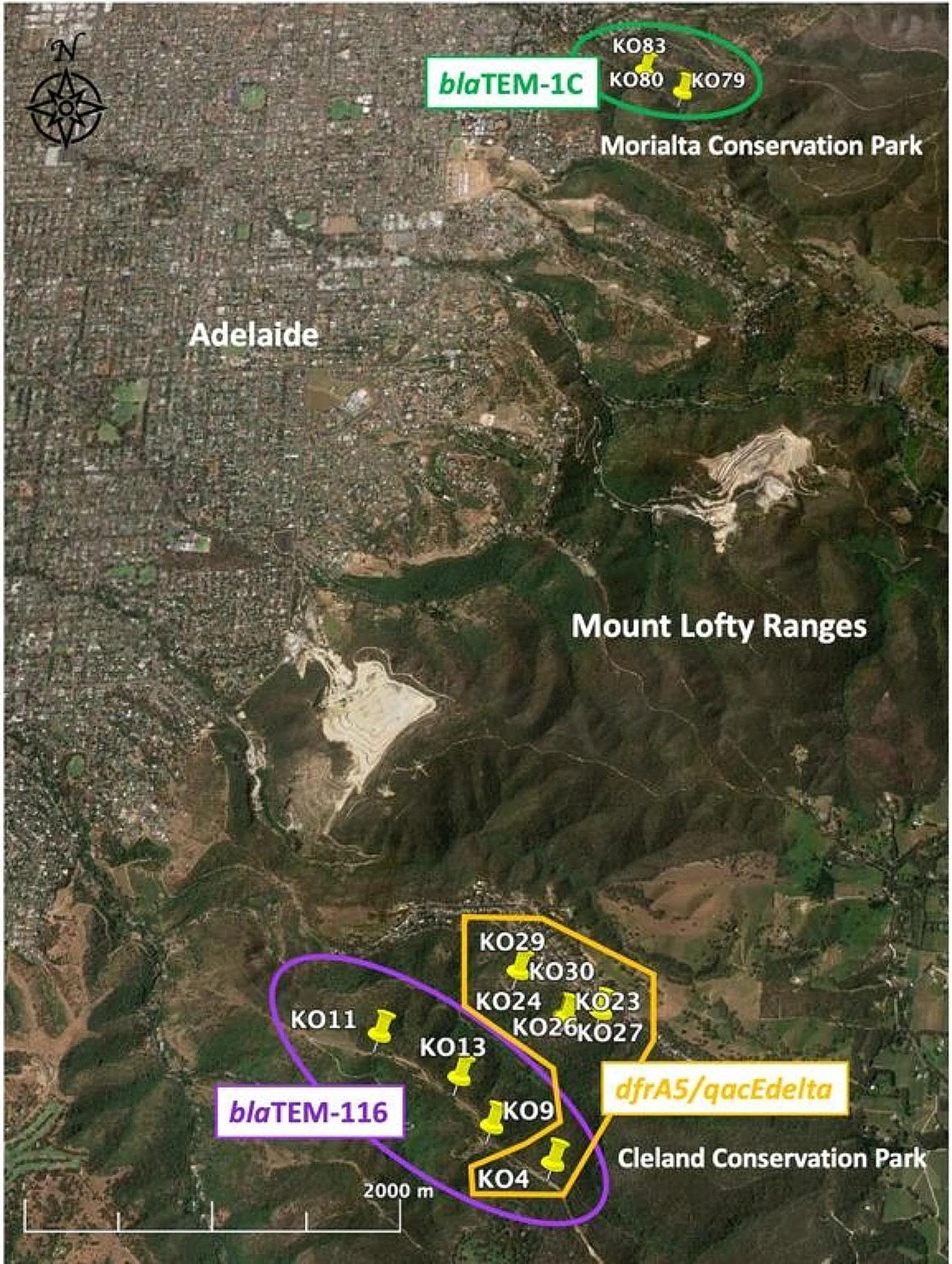
Satellite map showing the clustered GPS locations of all pre-fire wild-caught Mount Lofty Ranges koalas (Morialta Conservation Park 2018 and Cleland Conservation Park 2018) carrying *dfrA5/qacEdelta* integrons and *bla*TEM genes. GPS analysis and satellite map were constructed using Google Earth Pro (Version 7.3.6.9345, Google LLC, Menlo Park, USA).

Analysis of Global Positioning System (GPS) location data for all pre-fire (2018) wild-caught Mount Lofty Ranges koalas carrying *dfrA5/qacEdelta* integrons and *bla*TEM genes, found all *dfrA5/qacEdelta* positive koalas (Cleland, *n* = 7) were clustered within 1.05 km of each other and all *bla*TEM-116 positive koalas (Cleland, *n* = 4) were clustered within 1.2 km of each other, with both clusters located in the Cleland Conservation Park (Fig. 2). Notably, one koala (KO4) carried both a *dfrA5/qacEdelta* integron and a *bla*TEM-116 gene (Fig. 2). Similarly, all *bla*TEM-1 C positive koalas (Morialta, *n* = 3) were clustered within 325 m of each other in the Morialta Conservation Park (Fig. 2).

### **Association of Wildfires on*intI1*and*bla*TEM Occurrence**

**Fig. 3:**
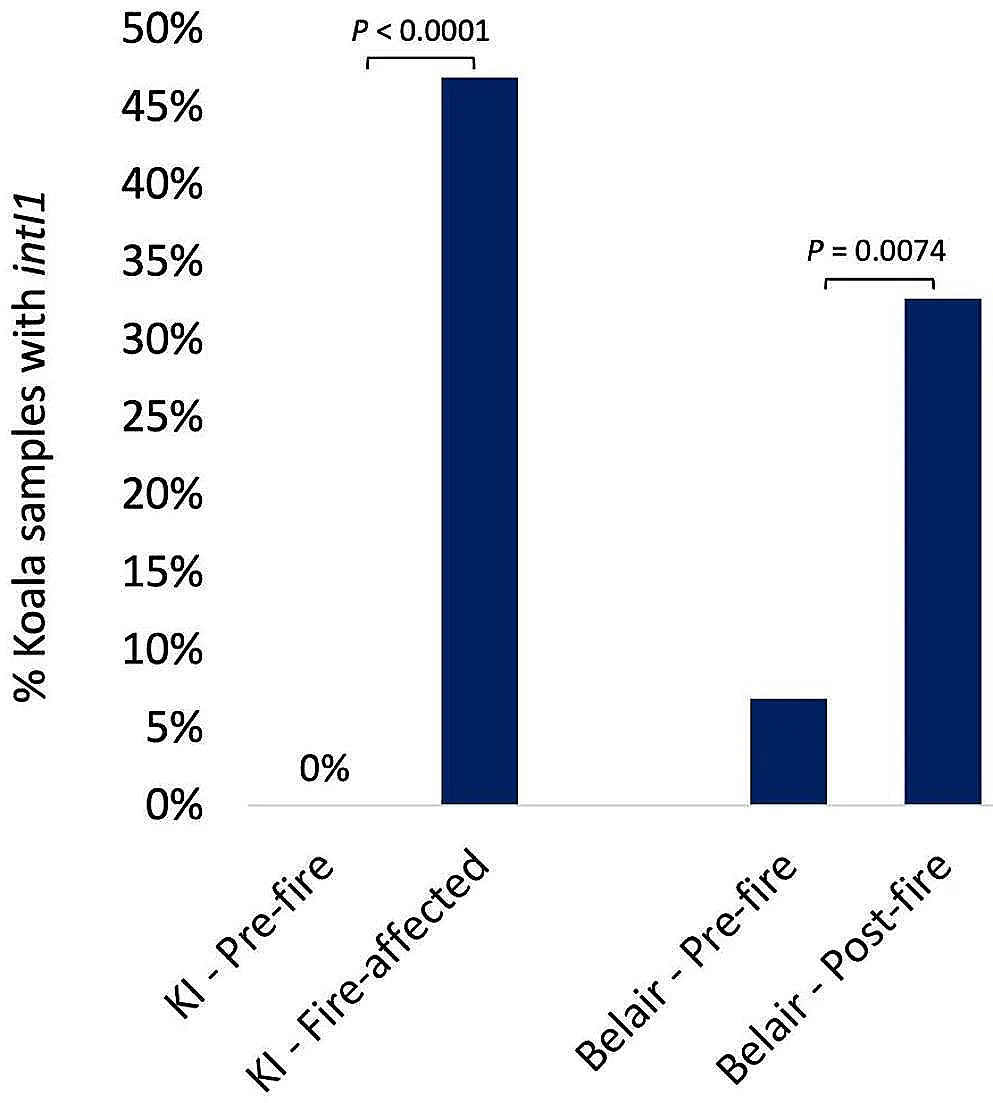
The occurrence of *intI1* and *bla*TEM genes in paired sampling events from koalas in two locations in South Australia, at two time points; Kangaroo Island (KI) pre-fire (2014–2017, *n* = 46) and fire-affected (2020, *n* = 32), and Belair National Park pre-fire (2018, *n* = 29) and post-fire (2022, *n* = 98). Statistical significance was indicated where *P* < 0.05 using Fisher’s exact test

For koalas on Kangaroo Island, the occurrence of *intI1* was significantly higher in fire-affected koalas that had entered into care (Kangaroo Island 2020) compared to wild pre-fire koalas (Kangaroo Island 2014–2017) (*P* < 0.0001) (Fig. 3). Similarly, koalas in Belair National Park, the occurrence of *intI1* was significantly higher in post-fire koalas (Belair 2022) compared to pre-fire koalas (Belair 2018) (*P* = 0.0074) (Fig. 3).

The *bla*TEM gene was only detected in two pre-fire samples (Cleland 2018 and Morialta 2018) (Table 1), of which, neither were re-sampled after the fire 2019/2020 fire season, as such, the association of fire and *bla*TEM occurrence in koalas could not be tested.

**Fig. 4:**
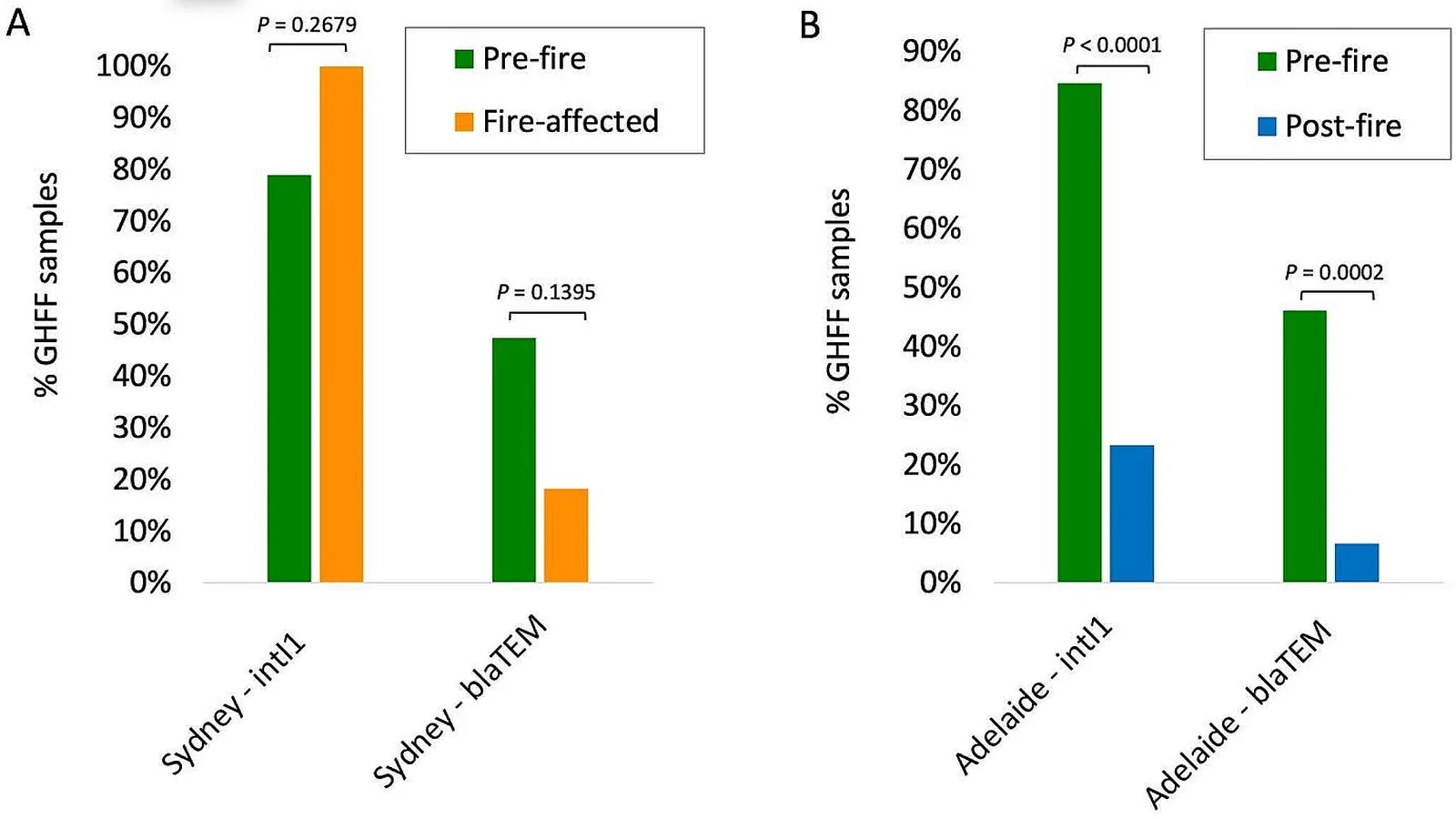
Occurrence of *intI1* and *bla*TEM genes in paired sampling events collected from Pteropid bat pups in the same locations at different time points. **(a)** Sydney pre-fire (2019, *n* = 19) and fire-affected (2020, *n* = 11). **(b)** Adelaide pre-fire (2018–2019, *n* = 52) and post-fire (2021, *n* = 30). Statistical significance was indicated where *P* < 0.05 using Fisher’s exact test

To determine the association of fire-season on *intI1* and *bla*TEM occurrence in bat pups, paired samplings collected from the same location at different time points, were evaluated for statistical difference (Fig. 4a and b). In bat pups in Sydney (New South Wales), the occurrence of both *intI1* and *bla*TEM were not significantly different in fire-affected bats (Sydney 2020) compared to pre-fire bats (Sydney 2018–2019) (*P* > 0.05) (Fig. 4a). In contrast, bats pups in Adelaide (South Australia), the occurrence of *intI1* and *bla*TEM were both significantly higher in pre-fire bats (Adelaide 2018–2019) compared to post-fire bats (Adelaide 2021) (*P* < 0.05) (Fig. 4b).

### ***intI1*and*bla*TEM Occurrence in Wild and In-Care Samples**

**Fig. 5:**
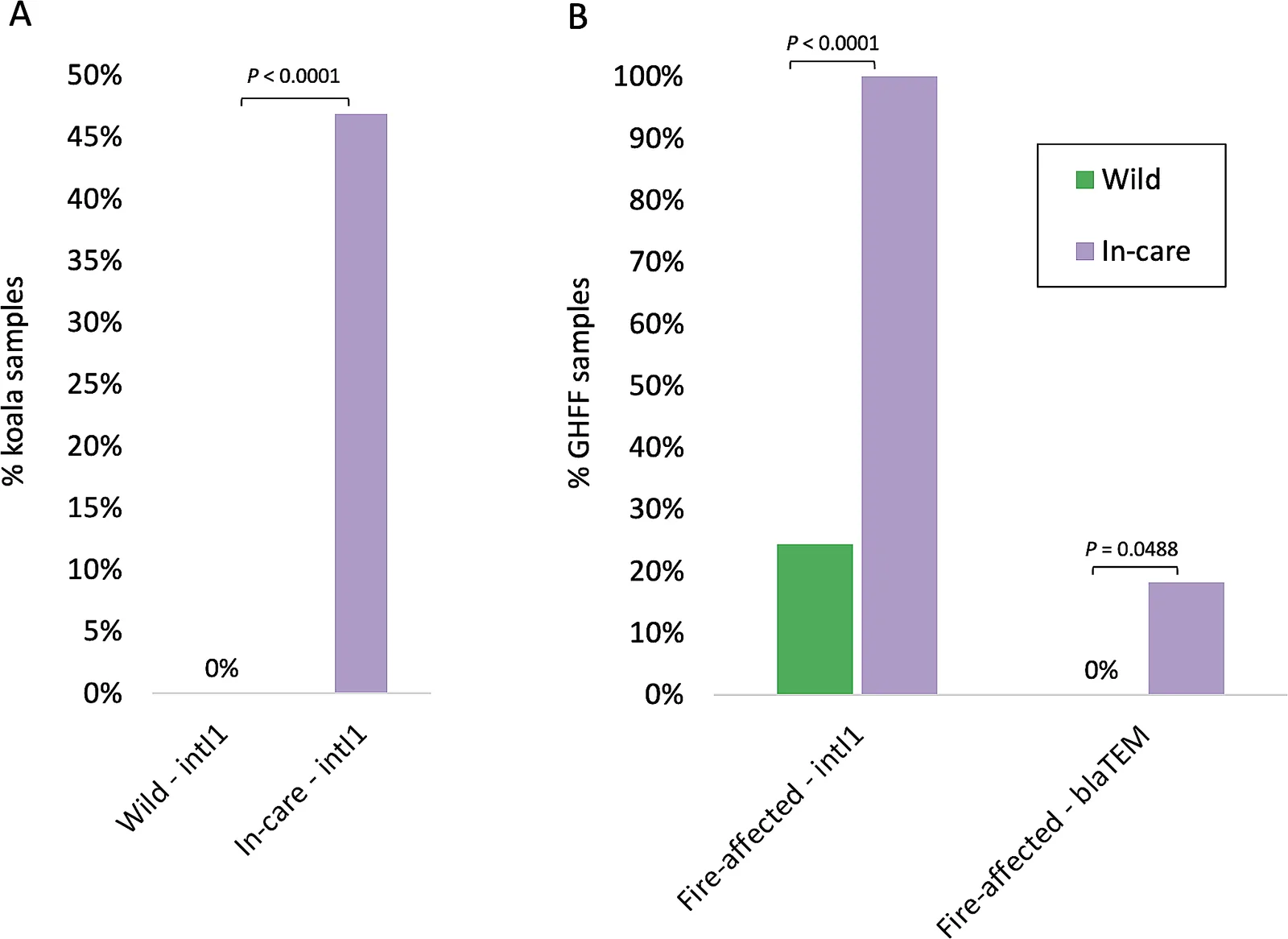
Occurrence of *intI1* and *bla*TEM genes in animals in-care compared to animals in the wild **a**. Kangaroo Island koalas (Wild, 2014–2017, *n* = 46. In-care, 2020, *n* = 32). **b.** Fire-affected Pteropid bat pups (Wild, Bega 2019–2020, *n* = 37. In-care, Sydney 2020, *n* = 11). Statistical significance was indicated where *P* < 0.05 using Fisher’s exact test

The association of entry into care on *intI1* and *bla*TEM occurrence in wild koalas and bat pups is significantly higher in both species in-care (Fig. 5). For koalas from Kangaroo Island, the occurrence of *intI1* was significantly higher (*P* < 0.0001) in koalas in-care (Kangaroo Island 2020) compared to wild koalas (Kangaroo Island 2014–2017), while *bla*TEM was not detected in either wild or in-care samples (Fig. 5a). For fire-affected bat pups, the occurrence of *intI1* and *bla*TEM were both significantly higher (*P* < 0.05) in bats in-care (Sydney 2020) compared to wild bats (Bega 2019–2020) (Fig. 5b).

### **Antibiotic Administration and Occurrence of*intI1*and*bla*TEM**

Treatment records were available for 27 of 32 Kangaroo Island koalas in-care and for all 118 bat pups in-care. Five Kangaroo Island koalas in-care (Kangaroo Island 2020) and three pre-fire Adelaide bat pups in-care (Adelaide 2018–2019), received antibiotics prior to the collection of samples (Supplementary Data 1). The five koalas received amoxicillin +/- clavulanic acid, with three of five also receiving enrofloxacin, and the three bat pups received amoxicillin + clavulanic acid (Supplementary Data 1). In the Kangaroo Island koalas, the occurrence of *intI1* was not significantly different in koalas which received antibiotics (3 of 6, 50.0%) compared to those that did not receive antibiotics (10 of 21, 47.6%) (*P* = 1.0000). Similarly, for the Adelaide bat pups, there was no significant difference in the occurrence of *intI1* in bats which received antibiotics (2 of 3, 66.7%) compared to those that did not receive antibiotics (42 of 49, 85.7%) (*p* = 0.4007). While *bla*TEM carriage was higher in the Adelaide bat pups which received antibiotics (3 of 3, 100%) compared to those that did not receive antibiotics (26 of 49, 53.1%), the difference was not statistically significant (*P* = 0.2455).

## Discussion

The frequency of the *intI1* gene in koalas and bat pups in this study is consistent with previous reports of *intI1* detected in other Australian wildlife species, including the little penguin (*E. minor*) [26], the brush-tailed rock-wallaby (*P. penicillate*) [25], Australian marine mammals [4] and adult grey-headed flying foxes (*P. poliocephalus*) [14]. The diversity of class 1 integrons containing ARGs in koala and bat pups is also consistent with those found in other Australian species, particularly, the high frequency of ARGs conferring resistance to aminoglycosides (*aadA*) and trimethoprim (*dfrA*) [4, 14, 25, 26].

This study is the first to report the presence of *bla*TEM genes in the faecal microbiome of wild koalas. Although the overall occurrence of *bla*TEM in koala samples was low (2.6%), for two locations the occurrence was 4- to 5-fold higher than the average (10.3% and 13.0%). The detection of three *bla*TEM gene variants in 25.2% of bat pups is consistent with previous reports of *bla*TEM genes in *Escherichia coli* from grey headed flying fox adults and pre-fire pups from Adelaide [6, 7]. The detection of an amoxicillin resistance gene (*bla*TEM) in koala and bat pup faecal microbiomes is highly concerning given that amoxicillin is a common antibiotic administered to both these species [29].

The bacterial species carrying class 1 integrons and *bla*TEM genes in koala and bat pup microbiomes were not identified as part of this study and therefore the changing patterns in AMR carriage cannot be linked to bacterial species dynamics. In a previous study where *E. coli* was cultured from the pre-fire Adelaide bat pup faecal samples used in this study (Adelaide 2018–2019), the *dfrA17/aadA5/IS26* class 1 integron was associated with a multidrug resistant *E. coli* strain ST48 O4:H26 [7] and the *bla*TEM-33 gene was associated with the beta-lactam resistant *E. coli* strain ST2144 O166:H49 [7].

The rare class 1 integron, *aadB/ORFX/OXA-2—aadA1/qacEdelta*, detected in a bat pup under care in a wildlife hospital, matched a single entry in GenBank, a Gram-positive isolate (*Corynebacterium amycolatum)* [57]. *Corynebacterium amycolatum* is an opportunistic pathogen associated with urinary tract infections in domestic animals [58, 59] and extraintestinal infections, including blood, endocarditis and peritonitis, in human patients [60, 61]. Notably, class 1 integrons are typically associated with Gram-negative bacteria and rarely with Gram-positive species [62]. Future work to determine if bat pups are carrying an antimicrobial resistant strain of *C. amycolatum* associated with integron carriage is required.

The clustering of the *dfrA17/aadA5/IS26* class 1 integron and the *bla*TEM-33 gene in specific cohorts of bat pups indicates that they acquired these ARGs from the same environmental source and/or via direct transmission of bacterial strains between co-housed bats as observed previously [7, 16, 63]. In koalas, *bla*TEM-1 C, *bla*TEM-116 and *dfrA5/qacEdelta* appear to also be acquired by wild koalas from the same environmental source given that the GPS locations of wild-caught koalas carrying three ARGs formed three distinct geographical clusters. Direct transmission between koalas is also possible, but less likely compared to potential for transmission between bat pups, given that koalas do not have frequent interactions, except during the breeding season [63, 64].

There was no significant association between antibiotic administration and carriage of *intI1* or *bla*TEM genes in either koalas or bats, however, this may be due to the small sample sizes of koalas and flying foxes which received antibiotics prior to sampling. Concerningly, the three bat pups which received amoxicillin + clavulanic acid were carrying *bla*TEM genes, of these, two were *bla*TEM-33 which confers resistance to amoxicillin + clavulanic acid and one pup carried *bla*TEM-1B which confers resistance to amoxicillin. Amoxicillin and trimethoprim are frequently used to treat infections in koalas [29] and multiple variants of *bla*TEM and *dfrA* genes (confer resistance to amoxicillin and trimethoprim respectively) were detected in koalas in this study. The presence of ARGs in wildlife presents a two-fold issue. Firstly, treatment failure may result from unresolved infection with antibiotic resistant bacteria and poor rehabilitation outcome. Secondly, antibiotic administration has the potential to promote the horizontal transfer of ARGs between bacterial species, and risk the emergence of new antimicrobial resistant pathogens [65]. It is imperative that antibiotics are used judiciously, and consideration is given to potential AMR carriage when treating fire-affected koalas and bat pups in-care. Overall, these findings reinforce the need for adherence to antimicrobial stewardship prescribing guidelines [66].

The substantial increase in carriage of *intI1* from zero detection in pre-fire koalas to 46.8% in fire-affected koalas in-care, indicates that koalas being driven into care by wildfires is associated with a higher carriage of class 1 integrons. This finding is consistent with previous reports of a higher prevalence of AMR in wildlife in rehabilitation environments, compared to their free-living counterparts [8, 14, 15]. *IntI* carriage in wild koalas in a fire-impacted environment (Belair National Park), and that did not enter care, was significantly higher in post-fire koalas (32.7%) compared to pre-fire koalas (6.9%), suggesting that wildfires are associated with higher carriage of class 1 integrons at this location.

In contrast to koalas, there was no significant association between wildfires and the carriage of *intI1* or *bla*TEM genes in pre-fire bat pups compared to fire-affected pups. There was a high prevalence of *intI1* and *bla*TEM in pre-fire pups from Sydney in 2018–2019 indicating that increased AMR carriage is also driven by non-fire factors, which may explain why there was no significant increase in *intI1* and *bla*TEM carriage by fire-affected pups. Further, the bats themselves were less likely to be directly impacted by fire unlike koalas. While this study examined fire as a driver of AMR dynamics, the sampling design was opportunistic. Emergency preparedness and study design tailored to wildlife emergencies would support understanding the association between fire and AMR, and the additional risks this poses for wildlife health and conservation. In agreement with koalas, fire-affected bat pups that entered care had a significantly higher carriage of both *intI1* and *bla*TEM genes compared to wild fire-affected pups. These findings further indicate that wildfires are shifting the dynamics of AMR in wildlife when they are driven into care following catastrophic wildfire events.

Multiple intertwined factors can drive acquisition of AMR by fire-affected wildlife that enter care; (1) increased pre-entry exposure to sources of antimicrobial resistant bacteria in fire-impacted environments, for example, via drinking from contaminated water sources [67–69], (2) transmission through close connectivity and exposure to wildlife already carrying AMR within wildlife hospital and rehabilitation environments [8, 14, 63], (3) increase in AMR frequency through antibiotic administration to wildlife in-care that may also exacerbate transmission between co-housed wildlife [8, 16, 65] and (4) post-entry exposure to antimicrobial resistant bacteria in human dominated environments [24]. Additionally, previous work has shown that other climate change impacts, including heat stress events in grey headed flying foxes (Pteropid bats), also drives increased prevalence and diversity of AMR in wildlife [7].

Climate change induced extreme weather events including, droughts and heat waves, are predicted to increase over time, and consequently, drive an increased risk of extreme wildfires occurring [36, 70]. Predictions made in the Garnaut Climate Change Review published in 2008 stated that the effect of climate change will be evident in Australia by 2020 with the occurrence of extreme wildfires and earlier and longer fire seasons [71]. Australia will continue to experience extreme weather and wildfires unless the climate crisis is alleviated [71]. These extreme climate change events and wildfires will drive increasing numbers of Australia’s native wildlife into care and rehabilitation [40, 49]. Furthermore, wildfire associated habitat loss and food shortages will continue to impact many threatened species, including koalas and the grey headed flying fox (Pteropid bats), potentially pushing them closer to extinction, unless actions are taken to reduce the risks of future extreme wildfires occurring [32, 38, 72].

While we have investigated the association between the impacts of wildfires on AMR dynamics in Australian wildlife, the escalating risk of wildfires around the globe, and some in critical wildlife habitat [35, 36], presents wider significance for AMR stewardship and ecology in wildlife.

## Electronic Supplementary Material

Below is the link to the electronic supplementary material.

Supplementary Material 1

## Data Availability

All data for individual koalas and flying foxes is provided in a supplementary file (Supplementary Data 1). Representative sequences for intI1, gene cassette arrays and blaTEM genes have been uploaded to GenBank under accession numbers OR095837-OR095856 (Supplementary Data  1).
